# PM_2.5_, Population Exposure and Economic Effects in Urban Agglomerations of China Using Ground-Based Monitoring Data

**DOI:** 10.3390/ijerph14070716

**Published:** 2017-07-03

**Authors:** Yonglin Shen, Ling Yao

**Affiliations:** 1 College of Information Engineering, China University of Geosciences, Wuhan 430074, China; shenyl@cug.edu.cn; 2 State Key Laboratory of Resources and Environmental Information System, Institute of Geographic Sciences and Natural Resources Research, Chinese Academy of Sciences, Beijing 100101, China; 3 Jiangsu Center for Collaborative Innovation in Geographical Information Resource Development and Application, Nanjing Normal University, Nanjing 210023, China

**Keywords:** fine particulate matter, population exposure, population-weighted mean, urban agglomeration

## Abstract

This paper adopts the PM_2.5_ concentration data obtained from 1497 station-based monitoring sites, population and gross domestic product (GDP) census data, revealing population exposure and economic effects of PM_2.5_ in four typical urban agglomerations of China, i.e., Beijing-Tianjin-Hebei (BTH), the Yangtze River delta (YRD), the Pearl River delta (PRD), and Chengdu-Chongqing (CC). The Cokriging interpolation method was used to estimate the PM_2.5_ concentration from station-level to grid-level. Next, an evaluation was conducted mainly at the grid-level with a cell size of 1 × 1 km, assisted by the urban agglomeration scale. Criteria including the population-weighted mean, the cumulative percent distribution and the correlation coefficient were applied in our evaluation. The results showed that the spatial pattern of population exposure in BTH was consistent with that of PM_2.5_ concentration, as well as changes in elevation. The topography was also an important factor in the accumulation of PM_2.5_ in CC. Moreover, the most polluted urban agglomeration based on the population-weighted mean was BTH, while the least was PRD. In terms of the cumulative percent distribution, only 0.51% of the population who lived in the four urban agglomerations, and 2.33% of the GDP that was produced in the four urban agglomerations, were associated with an annual PM_2.5_ concentration smaller than the Chinese National Ambient Air Quality Standard of 35 µg/m^3^. This indicates that the majority of people live in the high air polluted areas, and economic development contributes to air pollution. Our results are supported by the high correlation between population exposure and the corresponding GDP in each urban agglomeration.

## 1. Introduction

Fine particulate matter (also known as PM_2.5_ or respirable particles), which is suspended in the atmosphere with an aerodynamic diameter no more than 2.5 microns, has been recognized as a serious environmental concern due to its significant adverse effects on human health, climate biogeochemical cycle, and atmospheric chemistry [1,2]. Negative health effects are important consequences of population exposure to PM_2.5_, which makes exposure information essential for policy-makers to develop appropriate risk control policies [3].

In previous studies worldwide, the characteristics, chemical compositions, sources, and formation mechanism of PM_2.5_ have been researched [4,5], and conclusions were made that pollution arose at several levels of scale, e.g., a sampling site scale for traffic non-exhaust and re-suspended dust sources, an urban scale for combustion and industrial sources, and a regional scale [6]. Moreover, terrain conditions are one of the most important factors that affect PM_2.5_ concentration distribution [7]. Yao and Lu [8] found that the spatial distribution of annual average PM_2.5_ concentration coincides with China’s three gradient terrains. In China, many mega-cities have been investigated, e.g., Beijing [9,10], Shanghai [11], Guangzhou [12], Nanjing [13,14], Chengdu [15], Shenyang [16], and Changchun [17]. Furthermore, studies conducted in recent years have also focused on a regional scale, such as urban agglomeration. Urban agglomeration is an extended city or town area comprising the built-up area of one mega-city or more, with surrounding suburbs linked by continuous urban areas. Shimou [18] reviewed the differences among urban agglomerations in America, Europe, and China, and concluded that strict administration systems and regulations made the development much more difficult if a city tried to expand beyond their political borders in China. Zhao et al. [19] analyzed the chemical compositions, seasonal variations, and regional pollution events of PM_2.5_ in the Beijing-Tianjin-Hebei (hereinafter, BTH, also known as Jing-Jin-Ji), China. Moreover, other urban agglomerations of China were also examined, including the Yangtze River delta (YRD) [20], Pearl River delta (PRD) [21,22], and Chang-Zhu-Tan [23]. A comparison between cities and urban agglomerations has been conducted; Chan and Yao [24] presented an overview of air pollution in China and provided a detailed comparison between Beijing, Shanghai and the PRD.

To better quantify the adverse health impacts of PM_2.5_, population exposure estimates that can better incorporate the spatial variability of PM_2.5_ concentrations are required [1]. Aleksandropoulou and Lazaridis [25] evaluated the population exposure to PM_2.5_ in outdoor environments in the metropolitan areas of Greece from 2001 to 2010. Pant et al. [26] estimated the population exposure to PM_2.5_, and assessed the representability of ambient air quality monitoring stations to serve as surrogates for population exposure in New Delhi, India. Requia et al. [3] evaluated the spatial patterns of link-based PM_2.5_ emissions and subsequent human exposure in a large Canadian metropolitan area. Furthermore, evaluations have also been conducted in mega-cities of China, like Beijing [27,28]. Population of exposure to pollutant can be estimated either with satellite images or with ground-based monitoring data, where the former use satellite-derived aerosol optical depth (AOD) as a proxy for PM_2.5_ [1]. The variations of population exposure to PM_2.5_ across the PRD [29] and mainland China [30] have been stated by using the 0.1° × 0.1° moderate resolution imaging spectroradiometer (MODIS) satellite data. However, the accuracy of AOD-based PM_2.5_ retrieval methods may vary significantly due to seasons and locations [31,32].

In this study, we focused on the assessment of population exposure and economic effects on PM_2.5_ in typical urban agglomerations of China, i.e., BTH, YRD, PRD and Chengdu-Chongqing (hereinafter, CC, also known as Cheng-Yu). Station-based PM_2.5_ datasets across the four urban agglomerations were used, and criteria including population-weighted mean, cumulative percent distribution, and correlation coefficient were employed in our evaluation. This provided a unique opportunity for us to study the impact of grid-level PM_2.5_ data in exposure estimates for regional populations, as well as to further validate the economic effects on PM_2.5_ concentrations.

## 2. Materials and Methods

### 2.1. Study Area

**Figure 1:**
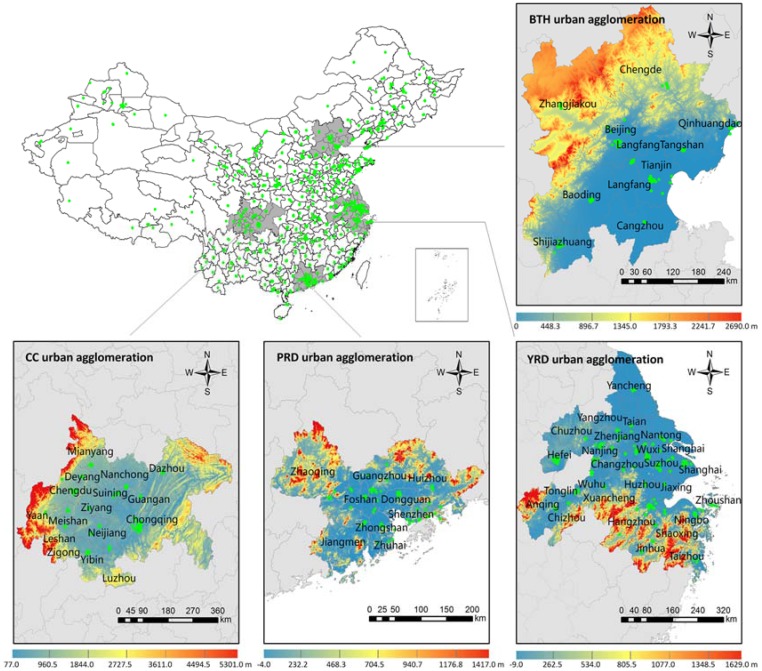
The location of the study areas and stations for PM_2.5_ monitoring. The color scale represents the elevation variability across the urban agglomeration.

The spatial domain of this study included the four most typical urban agglomerations of China, BTH, YRD, PRD and CC. In 2014, the population of the study areas accounted for 26% of the total in China, and the gross domestic product (GDP) of these regions accounted for 43.8% of the national GDP. The locations of the study areas are presented in Figure 1.

The BTH is the biggest urbanized region in northern China, which was born out of political pressure rather than economic prosperity. Ten cities including Beijing, Tianjin, Baoding, Langfang, Tangshan, Qinhuangdao, Shijiangzhuang, Zhangjiakou, Chengde and Cangzhou in the BTH urban agglomeration are officially recognized in the government publications.

The YRD region is the most economically developed and rapidly urbanizing city cluster in East China. It is located on the east coast of China bordering the East China Sea. The newest YRD urban agglomeration consists of Shanghai, Nanjing, Wuxi, Changzhou, Suzhou, Nantong, Yancheng, Yangzhou, Zhenjiang, Taizhou, Hangzhou, Ningbo, Jiaxing, Huzhou, Shaoxing, Jinhua, Zhoushan, Taizhou, Hefei, Wuhu, Ma’anshan, Tongling, Anqing, Chuzhou, Chizhou, and Xuancheng.

The PRD, which is currently known as the Guangdong-Hong Kong-Macau Greater Bay area, is situated in South China adjacent to the South China Sea, which is considered as one of the country’s chief economic regions and manufacturing centers. The PRD urban agglomeration generally comprises Guangzhou, Shenzhen, Zhuhai, Foshan, Dongguan, Zhongshan, Jiangmen, Zhaoqing, Huizhou, Hong Kong and Macau. As there is no ground-based monitoring data in Hong Kong and Macau, they were not considered in this study.

The CC is a national level urban agglomeration that leads the development of West China. It consists of Chongqing, Chengdu, Zigong, Luzhou, Deyang, Mianyang, Suining, Neijiang, Leshan, Nanchong, Meishan, Yibin, Guang’an, Dazhou, Ya’an and Ziyang, among which Chongqing and Chengdu are the core cities.

### 2.2. Datasets

The primary data used in this study were station-based PM_2.5_ concentrations, population census data, GDP census data and a 30 × 30 m digital elevation model (DEM). Annual PM_2.5_ concentration data were obtained from the China National Environmental Monitoring Center (CNEMC) website [33]. By the end of 2014, approximately 1497 monitoring sites had been established to report the overall air quality in China. The population and GDP census data were derived from the National Earth System Science Data Sharing Infrastructure [34] with 1 × 1 km sized grids. The census data showed a high density of people living in and around the major urban areas.

The original PM_2.5_ concentration data were at discrete points, i.e., at fixed monitoring stations. To present a localized representation of population exposure to PM_2.5_ and economic effects on it, we used a geographic information system based grid mapping where grids of 1 × 1 km were applied to PM_2.5_ data. Thus, the spatial interpolation method was applied to estimate the concentration of pollutants from station-level to grid-level to characterize concentrations across the entire area. In this study, we used Cokriging method incorporated with DEM data to calculate the pollutant concentration at each grid from the measured data at 1497 monitoring stations. Cokriging is a multivariate extension of kriging where the auxiliary information (in this case, DEM data) is incorporated in the estimation at unsampled locations by accounting for spatial correlations of the primary variable with secondary attributes [35]. The mapping processes divided regions of the BTH, YRD, PRD and CC into 146,598, 166,151, 36,103 and 208,879 available grid cells, respectively. Next, the processes of population exposure and the economic effects of urban agglomerations on those grid cells were analyzed by mathematical methods.

### 2.3. Estimation of Population Exposure

The population exposure was estimated both at the grid-level and region-level (i.e., at the urban agglomeration level). Population exposure at the grid-level was proposed as an important indicator to measure the population exposure to PM_2.5_ air pollution, which requires both population distribution data and PM_2.5_ concentration distribution data. It can be calculated by,

(1) $$\psi_{i}=P_{i}C_{i}$$

where $\psi_{i}$ represents the i th grid of population exposure; i designates each computational cell in the domain; $P_{i}$ is the population at a given cell location; and $C_{i}$ is the particulate concentration in the same cell location.

At the urban agglomeration level, the population exposure was measured by the population-weighted mean PM_2.5_ [36]. Calculating the population weighted mean PM_2.5_ has implications for estimating the impact of air pollution on public health and providing insights into pollution mitigation policies for individual urban agglomeration and cross-regional collaboration. If the distribution of PM_2.5_ within an urban agglomeration concurs with the spatial patterns of population, the resulting population-weighted mean PM_2.5_ tends to be larger than its original PM_2.5_ value, and vice versa [37]. The population weighted mean PM_2.5_ (denoted by ϵ) is calculated as

(2) $$\epsilon =\frac{\sum_{i=1}^{n}\psi_{i}}{P_{o}}$$

where $P_{o}=\overset{n}{\underset{i=1}{\sum }}P_{i}$ is the total population in the domain of interest (in this case, an urban agglomeration); and n represents the total number of grids in the urban agglomeration.

### 2.4. Estimation of Cumulative Percentage Distribution

Meanwhile, this paper also calculated the cumulative PM_2.5_ concentrations in each study area, as cumulative percentage is a way of expressing frequency distribution. It calculates the percentage of the cumulative frequency within each interval, as much as relative frequency distribution calculates the percentage of frequency [27]. The cumulative percent distribution of population or GDP by annual PM_2.5_ concentrations is calculated as

(3) $$\gamma_{\tau }=\frac{\int_{0}^{\tau }P_{C<\tau }}{P_{o}}\times 100\%$$

where $\gamma_{\tau }$ represents the cumulative percent distribution of population or GDP by the PM_2.5_ concentration τ; τ designates a certain concentration of PM_2.5_; $\overset{\tau }{\underset{0}{\int }}P_{C<\tau }$ is the cumulative population or GDP under the condition that the PM_2.5_ concentration is less than τ, i.e., C<τ.

### 2.5. Correlations among PM_2.5_, Population, GDP and Population Exposure

To measure the effects of population growth and economic development on PM_2.5_ concentrations, correlation coefficients were used to quantify the correlation and dependence among PM_2.5_, population, GDP, and population exposure. The correlation coefficient (r) measures the linear correlation between two variables x and y, and has a value between +1 and −1, where 1 is total positive linear correlation, 0 is no linear correlation, and −1 is total negative linear correlation. For each urban agglomeration, r was counted at both grid-level and city-level.

(4) $$r=\frac{\sum_{i=1}^{n}\left(x_{i}-\bar{x}\right)\left(y_{i}-\bar{y}\right)}{\sqrt{\sum_{i=1}^{n}{\left(x_{i}-\bar{x}\right)}^{2}\sum_{i=1}^{n}{\left(y_{i}-\bar{y}\right)}^{2}}}$$

where n represents the number to variable; $x_{i}$ and $y_{i}\;$ are the ith of variables x and y, respectively; and $\bar{x}$ and $\bar{y}$ are the mean of variables x and y, respectively. If r is counted at the grid-level, then x and y represent the values of each grid of 1 × 1 km, and n represents the number of cells; and if r is counted at the city-level, then x and y represent the values of each city, and n represents the number of cities.

## 3. Results and Discussion

This study was performed in three parts: (1) analysis of the spatial patterns of PM_2.5_ concentrations in four major urban agglomerations of China; (2) evaluation of population exposure to PM_2.5_; and (3) evaluation of economic impacts on PM_2.5_.

### 3.1. Spatial Patterns of PM_2.5_ Concentration

**Figure 2:**
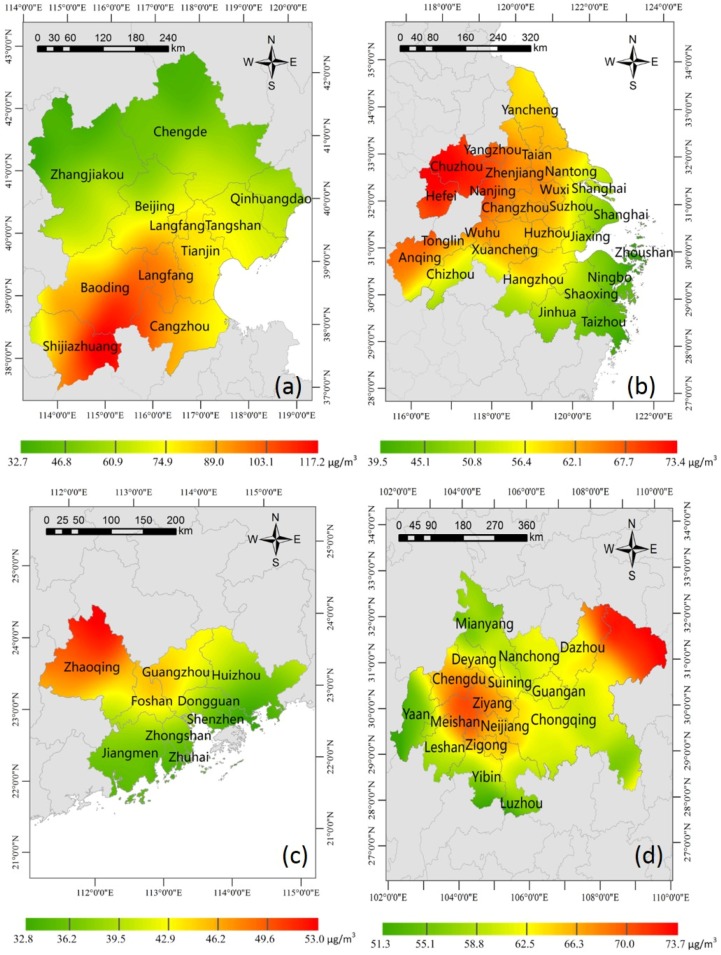
The spatial patterns of PM_2.5_ concentrations in the four urban agglomerations. (**a**) Beijing-Tianjin-Hebei; (**b**) Yangtze River delta; (**c**) Pearl River delta; and (**d**) Chengdu-Chongqing. Each subfigure has a different color scale to highlight the spatial variability across the corresponding urban agglomeration.

Urban PM_2.5_ originates mainly from sources such as coal combustion, traffic-related emissions, fugitive dust, biomass burning, agricultural activities and regional transported aerosols [38,39]. The World Health Organization (WHO) put forward an annual limit of 10 µg/m^3^ to mitigate its impact on human health [40]. The Chinese government adopted the interim Target 1 (IT1) of the air quality guidelines (AQG) established by the WHO. In 2012, the new Chinese National Ambient Air Quality Standard (NAAQS) was amended and issued by the Ministry of Environmental Protection of China (MEP). In the NAAQS, the annual PM_2.5_ concentration Grade II limit is 35 µg/m^3^ [41]. As shown in Table 1, the overall minimum and maximum of annual PM_2.5_ concentration in four urban agglomerations were 32.91 µg/m^3^ and 120.11 µg/m^3^, respectively, and were 3–12 times the standard set by the WHO of 10 µg/m^3^, and 1–3 times that of the NAAQS of 35 µg/m^3^. Additionally, the overall mean and standard deviation of PM_2.5_ concentration were 63.4 µg/m^3^ and 14.03 µg/m^3^, respectively. Figure 2 shows the spatial patterns of PM_2.5_ in the urban agglomerations. Since the four urban agglomerations had different maximum and the minimum concentrations (Table 1), the annual PM_2.5_ concentration of each urban agglomeration in Figure 2 was plotted in a subfigure with a different color scale to highlight the spatial variability across the corresponding urban agglomeration.

#### 3.1.1. Beijing-Tianjin-Hebei Urban Agglomeration

The PM_2.5_ concentration of the BTH urban agglomeration ranged from 37.21 µg/m^3^ to 120.11 µg/m^3^. The mean and standard deviation of PM_2.5_ concentration in BTH were 74.97 µg/m^3^ and 19.46 µg/m^3^, respectively, which were the highest in the four urban agglomerations (Table 1). As shown in Figure 2a, the higher PM_2.5_ concentrations were distributed in the southern areas of the BTH, and they decreased gradually from south to north. In particular, north Beijing and Tangshan were superior to their respective area in the south. The trends were consistent with the elevation changes shown in Figure 1. The air quality was relatively good in Zhangjiakou, Chengde, Qinhuangdao, and the corresponding PM_2.5_ concentration in these cities were 47.52 µg/m^3^, 59.45 µg/m^3^ and 66.06 µg/m^3^, respectively. The worst was Baoding with a concentration of 116.21 µg/m^3^, followed by Shijiangzhuang of 108.51 µg/m^3^.

Several previous studies have revealed the sources of PM_2.5_ pollutants in the BTH [19]. Zhao et al. [19] pointed out that BTH suffers from serious secondary pollution, and motor vehicle exhaust highly contribute to the PM_2.5_ concentration. From the perspective of transportation process, pollution sources can be placed into three categories: local emissions, local transportation and regional transportation. Furthermore, local emissions, local transportation, and regional transportation have all significantly contributed to high fine particle loadings in these areas [38].

The local emission sources for air pollution include power plants, domestic heating, and industrial, vehicular, and biogenic sources [24]. In Beijing and Tianjing, motor vehicle exhausted highly contributes to the annual PM_2.5_ concentrations. In addition to transportation, coal combustion was a vital factor affecting PM_2.5_ concentration, especially in Shijiazhuang and Chengde, where the PM_2.5_ pollution was dominated by coal combustion [19]. During winter, domestic heating emissions, mainly from coal combustion, contribute highly to the air quality degradation observed in these cities [42]. For example, domestic heating in Beijing usually starts in mid-November and ends in the following March, which is the major source of SO_2_ in winter [24].

Pollution source of local transportation is defined as the one that comes from other cities of the same urban agglomeration. Recent studies have shown that changes in atmospheric circulation (e.g., the weakened northerly winds and the development of inversion anomalies in the lower troposphere) may be an important reason for the increased haze pollution in northern China [43]. The prevailing wind in Beijing is from the north and the northwest, particularly in winter and spring, respectively, and the concentrations of air pollutants generally decrease with increasing wind speed [24].

Pollution source of regional transportation is the one that comes from areas beyond the urban agglomeration. Several studies have shown that the enhanced PM_2.5_ pollutants in BTH is not only due to the primary emissions from local sources (such as industrial, domestic and agricultural sources), but also the regional transportation contribution (e.g., from nearby Shandong and Henan Provinces) and secondary production. Furthermore, the climate of the BTH is characterized by stagnant weather with weak wind and relatively low boundary layer height, which is a favorable atmospheric condition for the accumulation, formation and processing of aerosols [39].

#### 3.1.2. Yangtze River Delta Urban Agglomeration

The PM_2.5_ concentration of the YRD urban agglomeration ranged from 38.51 µg/m^3^ to 75.43 µg/m^3^. The mean and standard deviation of PM_2.5_ concentration of the YRD were 59.24 µg/m^3^ and 7.68 µg/m^3^, respectively (Table 1). The mean concentration of PM_2.5_ in the YRD region was below average level (63.4 µg/m^3^), and lower than that of the BTH (74.97 µg/m^3^) and CC (63.32 µg/m^3^), but higher than that of the PRD region (43.39 µg/m^3^).

Industrial sources including power plants, other fuel combustion facilities, and non-combustion processes were the major contributors to the PM_2.5_ pollution in the YRD [44]. As shown in Figure 2b, the PM_2.5_ concentrations distributed in the northwest areas of the YRD were high, and decreased gradually from north and northwest to southeast. There are three capital cities (i.e., Hangzhou, Nanjing and Hefei) and a municipality (i.e., Shanghai) in the YRD. Hefei had the worst air quality (76.6 µg/m^3^), followed by Nanjing (68.23 µg/m^3^), Hangzhou (53.63 µg/m^3^), and Shanghai (49.59 µg/m^3^). Shanghai is located at downstream of the YRD, and the prevailing winds from the east carry clean air into the region [45,46]. The major abundant metal elements of the mass concentrations of PM_2.5_ in Shanghai, Nanjing, Hangzhou and Ningbo were crustal elements (e.g., Al, Ca, Fe and Mg) and trace metals (e.g., Zn, Pb, Cu, Cr, V and Ni) [20].

#### 3.1.3. Pearl River Delta Urban Agglomeration

The PM_2.5_ concentration of the PRD urban agglomeration ranged from 32.91 µg/m^3^ to 52.96 µg/m^3^. The mean and standard deviation of PM_2.5_ concentration of the PRD were respectively 41.34 µg/m^3^ and 4.54 µg/m^3^ (Table 1). The mean concentration of PM_2.5_ in the PRD was the lowest in the four urban agglomerations mainly because of fewer coal-based industries and good dispersion weather conditions [39]. Nevertheless, it was still four times the standard set by the WHO of 10 µg/m^3^, and higher than NAAQS at 6.34 µg/m^3^.

In the PRD, the level of secondary pollutants was higher than that of dusts and primary pollutants [47]. PM_2.5_ emissions mainly concentrated in Guangzhou, Foshan, Shenzhen and Dongguan, which posses advanced industry, high energy consumption, and dense population. However, the climate of the PRD is affected by monsoons. The prevailing winds are from the north in winter and the south in summer [1]. As shown in Figure 1, PRD is a low-lying area surrounding the Pearl River estuary, and the elevations of north PRD and parts of Jiangmen are relatively higher than that of other regions. Affected by the topography and climate characteristics, PM_2.5_ concentrations easily gather in the north of PRD in winter. As shown in Figure 2c, PM_2.5_ concentrations were lower near the coastline, and vice versa. Much higher PM_2.5_ concentrations were observed in the north of PRD, with orange and red colors showing annual PM_2.5_ concentrations higher than that of southern coastal areas.

#### 3.1.4. Chengdu-Chongqing Urban Agglomeration

The PM_2.5_ concentration of the CC urban agglomeration ranged from 51.15 µg/m^3^ to 73.97 µg/m^3^. The mean and standard deviation of PM_2.5_ concentration were 63.32 µg/m^3^ and 4.1 µg/m^3^, respectively (Table 1). Although its mean PM_2.5_ concentrations out of the four urban agglomerations was not the lowest, the standard deviation was. This suggested that concentration change in the whole urban agglomeration was relatively smooth and steady.

The maximum PM_2.5_ concentration in the CC was nearly two times the NAAQS of 35 µg/m^3^, which was partly due to its unique topographic condition [48]. Local PM_2.5_ emissions accompanied by low wind speed and high relative humidity conditions are major causes of visibility impairment in the Sichuan Basin [49]. As shown in Figure 2d, there are two hotspots in the CC: one is centered at the Sichuan Basin, where the capital city of Sichuan Province (i.e., Chengdu) and the downtown area of Chongqing are located, and another is centered at Dazhou.

Regarding the first hotspot (Figure 1), the elevation of cities there was lower than that of adjacent areas. Chengdu has typical basin climate characteristics, i.e., a precipitation period is basically from July to September with relatively high humidity, and static wind frequency, and there is atmospheric stability subject to neutral weather in winter. These special topographic and climatic conditions are important causes of the accumulation of PM_2.5_. Static wind and temperature inversion weather occur frequently, especially in winter and autumn, resulting in continuous heavy pollution [50]. Similarly, local contributors are likely to play a predominant role in the downtown of Chongqing. Influenced by its specific topographic conditions, Chongqing is located in the region of the lowest wind speed over China. For example, the annual average wind speed was between 0.9 m/s and 1.6 m/s from 1979 to 2007 [38]. The specific geographic and meteorological conditions favor the accumulation of regional and local pollutants, and stable weather conditions with low wind speed, low mixing heights, and high relative humidity can greatly enhance pollution levels [43].

In the second hotspot where the city of Dazhou is located, a variety of factors contributed to the higher concentration. The air pollution of Dazhou was mainly caused by the discharge of industrial dust and soot emissions from heavy industries (e.g., iron, steel, and cement), vehicular emissions, and urban fugitive dust. In addition, the topography of Dazhou is an objective factor, as it is surrounded by mountains on three sides in a narrow area. These anthropic and natural factors made Dazhou unusually prominent in the air pollution of the CC, thus, the eventual formation of one of the two hotspots.

### 3.2. Population Exposure to PM_2.5_

**Figure 3:**
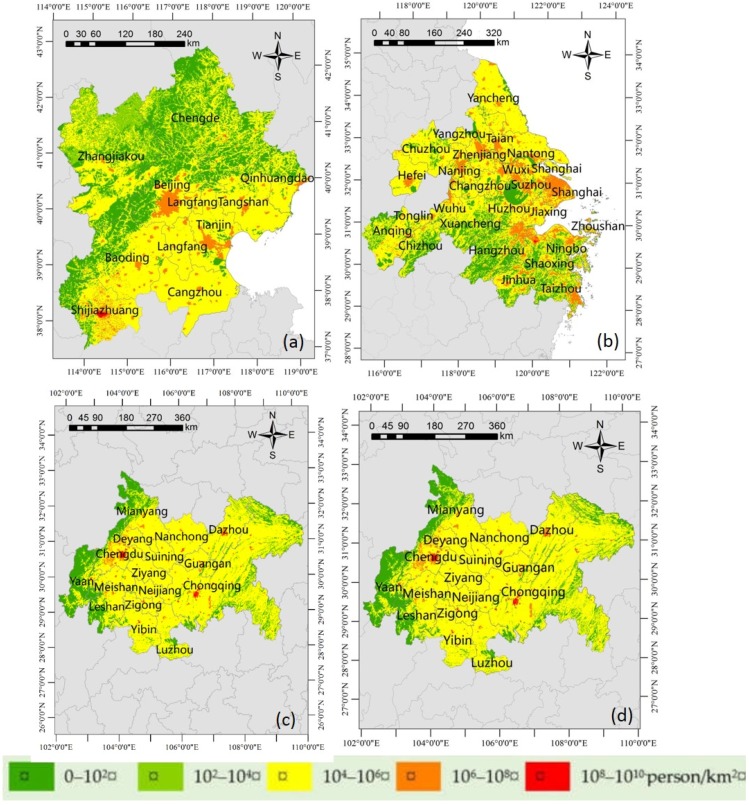
The population exposure to PM_2.5_ in the four urban agglomerations. (**a**) Beijing-Tianjin-Hebei; (**b**) Yangtze River delta; (**c**) Pearl River delta; and (**d**) Chengdu-Chongqing.

The total population of the four urban agglomerations was estimated at about 355 million, of which about 77.8 million reside in BTH, and 129.9, 31.4, and 115.9 respectively in YRD, PRD, and CC. A comparison of the spatial distributions of population exposure among the four urban agglomerations in Figure 3 showed that areas in the mega-cities were often associated with higher population exposure to PM_2.5_. For example, as shown in Figure 3a, a larger proportion of the population was exposed to high PM_2.5_ concentrations in Beijing, Tianjin, and Shijiazhuang, which are capital cities or municipalities in the BTH. Nevertheless, the spatial pattern of population exposure in the BTH was consistent with that of PM_2.5_ concentration, and both of them decreased gradually from south to north. On 1 April 2017, the Xiongan new area (also known as the Hung an District) was set up in Baoding, Hebei Province and was a key component of a massive mega region developing around Beijing, Tianjin, and Hebei. With further economic development of the region, it is expected that the population will rise sharply. As shown in Figure 2a, PM_2.5_ concentration of this area was quite high. As the population increasing, the population exposure to PM_2.5_ should be very serious. Thus, it is suggested that during the process of the district construction, much attention should be paid to environmental pollution control.

In the YRD as shown in Figure 3b, central area had the highest PM_2.5_ concentration, followed by the northern area, and the air quality in the south was the best. In the PRD, population density had a strong effect on population exposure. Therefore, the spatial pattern of the PM_2.5_ concentrations was opposite to that of the corresponding population exposure (Figure 3c). As mentioned above, the PM_2.5_ concentrations of PRD decreased from the north to the southern coastal areas; whereas the population exposure to PM_2.5_ was more serious in the southern coastal areas such as Guangzhou and Shenzhen, which are two major mega-cities situated in the PRD region. The population exposure distribution also showed that these two cities have the most important position in this urban agglomeration. Furthermore, the PRD region—especially the areas between the two mega-cities—has undergone rapid urbanization and many smaller towns like Dongguan with a population of about one million have been established in the last two decades. The air quality in those cities and towns has been deteriorating over the last decade [24], and the corresponding population exposure is very grim.

As shown in Figure 3d, the outside of the CC had a relatively low population exposure, and the middle part had a level of moderate severity except for the three hotspots, i.e., Chengdu, Chongqing, and Dazhou.

Based on the grid-level annual PM_2.5_ concentration and population census data in each urban agglomeration, the population-weighted mean of PM_2.5_ in the BTH, YRD, PRD, and CC urban agglomerations were 86.11 µg/m^3^, 58.32 µg/m^3^, 41.12 µg/m^3^ and 63.63 µg/m^3^, respectively (Table 1), which were approximately 2 to 4 times as high as the global population-weighted mean (20 µg/m^3^) [51]. The population-weighted mean of PM_2.5_ across all four urban agglomerations was 64.62 µg/m^3^. We found that the population-weighted mean of PM_2.5_ in the YRD, PRD, and CC were under 64.62 µg/m^3^, while that of BTH was far more than that.

In terms of the population-weighted mean of PM_2.5_ concentrations, the most polluted urban agglomeration was BTH and the least was PRD. For the BTH, the spatial average of PM_2.5_ concentration in 2014 was 74.97 µg/m^3^, while the corresponding population-weighted mean of PM_2.5_ was much higher at 86.11 µg/m^3^, which indicated that more people were living in highly polluted areas. Hence, the spatial average of the PM_2.5_ concentration would underestimate the mean pollution exposure, and the population weighted average would be a better indicator of the public exposure to PM_2.5_ pollution [1]. The only urban agglomeration with the population-weighted mean less than that of the spatial average was CC, which was indicative that more people were living in low pollution areas.

**Figure 4:**
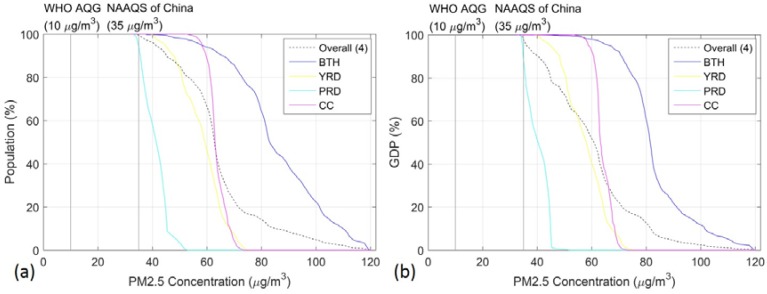
Cumulative percent distribution by annual average PM_2.5_ concentrations which is estimated from station-based monitoring data in 2014 (**a**) Cumulative percent distribution of population; (**b**) Cumulative percent distribution of GDP. The dashed line represents the computing on the overall four urban agglomerations.

Using the 1 × 1 km grid-level PM_2.5_ concentration, the cumulative percentage of the population (0–100%) in the four typical urban agglomerations of China are shown in Figure 4a. The results showed that the WHO AQG (10 µg/m^3^) for PM_2.5_ was exceeded by 100% of the population in the study area, while China’s NAAQS was exceeded by 99.49% of the population. Figure 4a also shows the cumulative percent distribution of population by annual PM_2.5_ concentration in BTH, YRD, PRD and CC urban agglomerations. Overall, only 0.51% of the population lived in the four urban agglomerations with an annual average PM_2.5_ concentration smaller than China’s NAAQS Grade II of 35 µg/m^3^, while the quantity was 5.8% for the PRD, and no one in the BTH, YRD and CC urban agglomerations lived under this guideline. It should be noted that all populations lived in the four urban agglomerations where the WHO AQG was exceeded.

However, there were some special cases including areas with higher PM_2.5_ concentration, but lower population density (e.g., industrial parks). This can be divided into two scenarios: (1) The industrial park is seriously polluted, but has fewer employees (the levels of population exposure can be directly indicated by Figure 3); and (2) the industrial park is seriously polluted and has many employees, but the vast majority of employees are migrants or live outside the same 1 × 1 km grid. The population exposure of this scenario can be measured by the population weighted mean of PM_2.5_.

### 3.3. Economic Effects to PM_2.5_

Fast-economic development and high energy consumption have led to record heavy haze pollution days in many regions of China [8,16]. The total GDP of the four urban agglomerations had been estimated at approximately 29.4 trillion, of which about 6.1, 13.3, 5.8, and 4.2 trillion were produced by BTH, YRD, PRD and CC, respectively.

Based on the grid-level annual PM_2.5_ concentration and GDP census data in each urban agglomeration, the cumulative percentage distribution of the GDP (0–100%) was estimated (Figure 4b). Overall, only 2.33% of the GDP was produced in the four urban agglomerations with annual average PM_2.5_ concentration lower than the China’s NAAQS Grade II of 35 µg/m^3^. And the quantity was 11.8% for PRD and none of the GDP in the BTH, YRD, and CC urban agglomerations was produced under this guideline. It should be noted that all GDP of the four urban agglomerations exceeded the WHO AQG (10 µg/m^3^).

We calculated the correlation coefficient between PM_2.5_ concentration and the corresponding population in each urban agglomeration with counting at the grid-level (denoted by $R_{pp}$, as shown in Table 2). $R_{pp}$ in the BTH, YRD, PRD and CC were 0.26, –0.08, –0.04, and 0.04 respectively, and overall for the four urban agglomerations was 0.03. All studied regions showed that PM_2.5_ concentration and population were independent. The correlation coefficient with counting at the city-level (denoted by $R_{pp}^{\prime }$), where the $R_{pp}^{\prime }$ in the BTH, YRD, PRD, and CC respectively were 0.13, –0.19, 0.08 and –0.02, respectively, and across the overall four urban agglomerations was –0.05. The results showed that population was not a direct factor influencing PM_2.5_ concentrations at local scale, i.e., at a 1 × 1 km grid-level or city-level. The uncorrelated relationship of PM_2.5_ concentrations with population was consistent with previous findings conducted in other places around the world [39,51,52].

The correlation coefficient between PM_2.5_ concentration and GDP at the grid-level (denoted by $R_{pg}$) also showed that there was no connection between the two variables. The weak correlation was partly due to transporting the pollution, and the impact of economic development on PM_2.5_ concentrations was revealed in larger spatial scales, i.e., bigger than a 1 × 1 km cell scale. At the city-level (denoted by $R_{pg}^{\prime }$), a good correlation coefficient of 0.69 and 0.6 were found in the BTH and PRD, respectively, and this indicated that the GDP of the internal city was a vital factor for the increase of PM_2.5_ concentrations in these two urban agglomerations. For example, previous studies had shown that the growth in GDP had increased the demand for vehicular transport, and exacerbated Beijing’s air pollution problems [24].

We also calculated the correlation coefficient between population exposure and the corresponding GDP in each urban agglomeration. Most values at both grid-level and city-level showed good correlation coefficients, except for the PRD at grid-level, which was only 0.38. The correlation coefficient of population and corresponding GDP at the grid-level also showed a weak dependence, and the value was 0.41. The spatial trends of population exposure and population were related closely as economic growth attracted a large migrant population into the PRD, and provided cheap labor for economic development. There were more than 23 million migrants living in the PRD in 2009, accounting for more than 40% of the total resident population in the region [29]. Most migrants were concentrated in Shenzhen, Guangzhou, Dongguan and Foshan; however, the population census data only related to the resident population, which decreased the correlation coefficient between population exposure and the corresponding GDP in the PRD.

## 4. Conclusions

In summary, the population exposure and economic effects on PM_2.5_ in BTH, YRD, PRD and CC urban agglomerations were evaluated. The station-based PM_2.5_ concentrations, population and GDP census data were used, as well as the population exposure estimated for each grid sized 1 × 1 km and for each urban agglomeration. The latter was measured by population-weighted mean PM_2.5_ and cumulative percent distribution. The economic effects on PM_2.5_ were also measured by cumulative percent distribution, as well as correlation coefficient.

PM_2.5_ concentrations were generally higher in the urban agglomeration of North China (e.g., the BTH) than those observed in the south (e.g., the YRD, PRD, and CC). Domestic heating and unfavorable meteorological conditions for pollution dispersion significantly contributed to high fine particle loadings in the BTH. PM_2.5_ concentrations in the BTH increased gradually from north to south, which were consistent with trends in elevation changes. PM_2.5_ concentrations in YRD and PRD also tended to be lower in the coastal regions than in the inland. There were two concentration hotspots in the CC, one centered at the Sichuan Basin and another at Dazhou. Furthermore, areas in mega-cities were often associated with higher population exposure to PM_2.5_, and the spatial pattern of population exposure in the BTH was consistent with the spatial pattern of PM_2.5_ concentrations. The highest PM_2.5_ concentration was in central YRD. The spatial distribution of PM_2.5_ concentrations was the opposite to that of the corresponding population exposure in the PRD. The population exposure outside of the CC was lower than that of the middle part. In terms of population-weighted mean of PM_2.5_ concentrations, the most polluted urban agglomeration was BTH, and the least was PRD. In terms of the cumulative percent distribution of population, only 0.51% of the population in the four urban agglomerations lived with an annual average PM_2.5_ concentrations smaller than the NAAQS standard. Only 2.33% of the GDP was produced in the four urban agglomerations with annual average PM_2.5_ concentration smaller than NAAQS. Generally, fast-economic development has led to serious population exposure to PM_2.5_ in most urban agglomerations.

In the future, multi-year series of PM_2.5_ concentrations will be used, and further studies will focus on inter-annual variations, seasonal changes, as well as the long-range spatial transport effects related to population exposure of PM_2.5_ concentrations, and the economic effects upon this.

## Figures and Tables

**Table 1:** Statistical information of the urban agglomerations.

Urban Agglomeration	$C_{min}$ ($\mu g/m^{3}$)	$C_{max}$ ($\mu g/m^{3}$)	$C_{mean}$ ($\mu g/m^{3}$)	$C_{std}$ ($\mu g/m^{3}$)	$C_{pwm}$ ($\mu g/m^{3}$)
BTH	37.21	120.11	74.97	19.46	86.11
YRD	38.51	75.43	59.24	7.68	58.32
PRD	32.91	52.96	41.34	4.54	41.12
CC	51.15	73.97	63.32	4.1	63.63
Overall	32.91	120.11	63.74	14.02	64.62

Notes: $C_{min}$, $C_{max}$, $C_{mean}$ and $C_{std}$ represent the minimum, maximum, mean and standard deviation of PM_2.5_ concentration in the domain of interest; and $C_{pwm}$ is the population-weighted mean of PM_2.5_ concentration.

**Table 2:** Correlation coefficient of PM_2.5_ concentrations, population, GDP, population exposure in urban agglomeration.

Urban Agglomeration	Grid-Level			City-Level		
	$R_{pp}$	$R_{pg}$	$R_{eg}$	$R_{pp}^{\prime }$	$R_{pg}^{\prime }$	$R_{eg}^{\prime }$
BTH	0.26	0.11	0.81	0.13	0.69	0.76
YRD	–0.08	–0.07	0.91	–0.19	–0.17	0.79
PRD	–0.04	–0.10	0.38	0.08	0.6	0.73
CC	0.04	0.03	0.88	–0.02	–0.08	0.92
Overall	0.03	–0.06	0.72	–0.05	0.28	0.64

Notes: $R_{pp}$, $R_{pg}$, and $R_{eg}$ respectively are the correlation coefficient between PM_2.5_ concentration and population; PM_2.5_ concentration and GDP; and population exposure and GDP, which are counted in the grid-level. While, $R_{PP}^{\prime }$, $R_{Pg}^{\prime }$, and $R_{eg}^{\prime }$ respectively are the correlation coefficient between PM_2.5_ concentration and population; PM_2.5_ concentration and GDP; and population exposure and GDP, which are counted in the city-level. The underscored values are highlighted to indicate the high dependence.
